# Surgical Wound Care Practices of Nurses in a Low‐Resourced Setting: An Observational Study

**DOI:** 10.1111/jocn.70445

**Published:** 2026-07-12

**Authors:** Gayani Priyangika Gamage, Sanjeewa Seneviratne, Josephine Lovegrove, Georgia Tobiano, Brigid Mary Gillespie

**Affiliations:** ^1^ School of Nursing and Midwifery Griffith University Southport Queensland Australia; ^2^ NHMRC Centre of Research Excellence in Wiser Wound Care Griffith University Southport Queensland Australia; ^3^ Department of Nursing and Midwifery, Faculty of Allied Health Sciences General Sir John Kotelawala Defence University Colombo Sri Lanka; ^4^ Department of Surgery, Faculty of Medicine University of Colombo Colombo Sri Lanka; ^5^ Herston Infectious Diseases Institute, Metro North Health Brisbane Queensland Australia; ^6^ School of Nursing, Midwifery & Social Work The University of Queensland, UQCCR Herston Queensland Australia; ^7^ Nursing and Midwifery Education and Research Unit, Gold Coast University Hospital Gold Coast Hospital and Health Service Southport Queensland Australia

**Keywords:** low‐resourced setting, practices, registered nurse, surgical wound care

## Abstract

**Aim:**

To observe and describe the surgical wound care practices of nurses in a Sri Lankan tertiary care hospital.

**Design:**

A cross‐sectional observational study.

**Methods:**

We observed surgical wound care practices of registered nurses (RNs) working in 13 general surgical wards of a 3000‐bed tertiary care hospital in Sri Lanka. An observation tool developed based on clinical practice guidelines (CPGs) and a chart audit tool were used for data collection. Field notes were taken to describe contextual details. A convenience sample of RNs and a consecutive sample of adult, postoperative patients were recruited. Patients were included once, while nurses were observed on multiple occasions. Descriptive statistics were used, and field notes were added to contextualise the data.

**Results:**

We observed wound care episodes across 250 postoperative patients with 63 RNs. Nurses' hand hygiene (HH) adherence was 58.4% (*n* = 146) before dressing changes and 43.2% (*n* = 108) after dressing changes. Wound care education was observed in only 22% (*n* = 55) of episodes. Documentation of wound care was noted only on 53 (21.2%) occasions. Field notes highlighted the lack of standard wound assessment and management protocols and resource constraints influencing the nurses' adherence to CPGs on many occasions.

**Conclusion:**

Our findings suggest that nurses' adherence to CPGs was limited, and there are unwarranted variations in nurses' surgical wound care practices. Unavailability of standard wound care protocols and resource constraints were also identified. These variations appear multifactorial, reflecting limitations in training, staffing, resources and the absence of standardised wound care protocols.

**Implications for the Profession and/or Patient Care:**

Targeted training programs should be conducted for nurses, and context‐sensitive wound management protocols should be introduced.

**Impact:**

As one of the first studies to describe nurses' surgical wound care practices in a resource‐limited country, these findings offer valuable insights into both practice and the contextual influences.

**Reporting Method:**

The Strengthening the Reporting of Observational Studies in Epidemiology guidelines.

**Patient or Public Contribution:**

No patient or public contribution.

## What Does This Paper Contribute to the Wider Global Clinical Community?

- This study provides empirical evidence of nurses' current surgical wound care practices in a resource‐limited setting.
- By including field notes, the study highlights the contextual insights and implementation challenges that influence nurses' unwarranted practices in a low‐resourced setting.
- These findings offer a foundation for developing targeted interventions that can influence the nurses' adherence to CPGs and contextual improvements needed for low‐resourced settings.

## 1 Introduction

Globally, over 313 million surgical procedures are performed per year (Meara et al. 2015) often involving a surgical wound. A surgical wound is an incision or cut into the skin formed under sterile conditions by a skilled professional during a surgical procedure (National Institute for Health and Care Excellence 2019). These wounds are vulnerable to various infectious and non‐infectious complications (Sandy‐Hodgetts et al. 2020) contributing to increased morbidity, prolonged hospital stays and higher healthcare costs worldwide (Berríos‐Torres et al. 2017). The most common complication associated with any surgery is surgical site infection (SSI) (Sandy‐Hodgetts et al. 2020). Results of one meta‐analysis indicated that the global 30‐day cumulative incidence of SSI was 11% (95% Confidence Interval [CI] 10%–13%) among general surgical patients between 2010 and 2021 (Gillespie et al. 2021). Another systematic review across 35 countries suggested that the pooled incidence of SSI in low‐ and middle‐income countries (LMIC) was 11.8 in 100 surgical procedures (Allegranzi et al. 2011). Comparatively, LMICs carry a disproportionately greater SSI burden, with one‐third of surgical patients globally developing an SSI (World Health Organization 2018). Thus, prioritising strategies that minimise surgical wound complications and support optimal wound healing is essential for improving patient outcomes.

## 2 Background

Surgical wound care is primarily carried out by nurses, in coordination with the multidisciplinary team (Gillespie et al. 2020). Providing wound care demands evidence‐based wound care practices, including appropriate dressing management, assessment and documentation and patient education (Ding et al. 2017; Lindsay et al. 2017). Over the past 25 years, different international clinical practice guidelines (CPGs) on the management or prevention of SSI have been developed and updated (Berríos‐Torres et al. 2017; Haesler and Carville 2023; National Institute for Health and Care Excellence 2019; World Health Organization 2018). Despite these recommendations, observational studies in high‐income countries have reported variable compliance with CPGs, including dressing management (e.g., hand hygiene and wound cleansing protocols) and documentation standards (Ding et al. 2017; Gillespie et al. 2020). The findings of previous studies have also suggested that nurses' wound care practices do not consistently align with evidence‐based protocols, which may increase the risk of surgical wound complications such as SSIs (Welsh 2018). However, much of this evidence is based on studies published between 2009 and 2017 and may not fully reflect current best practice. A recent integrative review of 36 studies indicated that acute care nurses predominantly prioritise technical dressing change tasks, with limited attention to other aspects of holistic surgical wound management (Gamage et al. 2025). Further, these review findings highlighted that nurses' practices are influenced by personal and organisational factors, as well as the scope of practice and collaboration between the multidisciplinary team. However, evidence on surgical wound care practices and the applicability of available CPGs in low‐resource settings remains limited, and no studies to date have used direct observations to capture nurses' actual clinical practice. There is a need to explore nurses' surgical wound care practices and the applicability of existing CPGs to LMICs, including Sri Lanka, using direct observation.

Sri Lanka is a lower‐middle‐income country, where the public sector delivers 95% of inpatient care (Ministry of Health Sri Lanka 2025). Although nurses manage a high volume of postoperative patients, as of April 2025, no published studies focus specifically on the surgical wound care practices of nurses in acute care settings in the Sri Lankan context. Given the critical role of nurses in surgical wound management, it is essential to explore how wound care is delivered in Sri Lankan hospitals. This gap highlights the need for systematic observation of wound care practices to understand current standards and identify areas for improvement. Observational research offers an opportunity to capture real‐world practices and contextual factors that influence care, which cannot be fully understood through self‐reported surveys alone (von Elm et al. 2014). By observing nurses' wound care practices in the largest tertiary care setting of Sri Lanka, this study aims to generate baseline evidence that can guide training initiatives, inform policy and ultimately improve patient outcomes in surgical care.

## 3 Aim

This study aimed to observe and describe nurses' surgical wound care practices for adult patients in the general surgical wards of a Sri‐Lankan tertiary care hospital.

## 4 Methods

### 4.1 Study Design

A cross‐sectional observational study was conducted with structured observations and chart audits (Vandenbroucke et al. 2007). Its reporting reflects the Strengthening the Reporting of Observational Studies in Epidemiology (STROBE) guidelines (von Elm et al. 2014).

### 4.2 Setting and Participants

This study was conducted in 13 general surgery wards across a 3000‐bed tertiary care hospital in Colombo District, Sri Lanka. In total, the general surgery wards had approximately 500 beds and employed 190 RNs. There were six general surgical theatres, each with two operating tables and one emergency general surgical theatre open around the clock, performing approximately 1000–1100 routine surgeries per month.

A convenience sample of eligible nurses and a consecutive sample of eligible postoperative patients (Table 1) were recruited. Patients were included only once via observation of a ‘wound care episode’ (defined as a series of activities undertaken by a nurse to manage the surgical wound of a patient). Some nurses were observed on multiple occasions due to the nature of the wound care process in the wards (i.e., the same nurse may perform wound care for multiple patients).

**TABLE 1:** Inclusion and exclusion criteria for nurses and patients.

	Nurses	Patients
Inclusion criteria	RNs Working in the permanent duty roster of the ward Willing to provide written consent to participate	Aged 18 years and older Had undergone any general surgery involving a surgical incision Receiving wound care either as an outpatient or inpatient in the respective ward Willing to provide written consent
Exclusion criteria	Having less than two months of experience as an RN RNs assisting with the surgical dressing change procedure	Undergone a laparoscopic surgery Patients > 30 days post‐surgery Patients deemed to be too unwell to understand and provide written informed consent by the ward nurse in charge

Abbreviation: RNs, registered nurses.

### 4.3 Study Instruments

#### 4.3.1 Observation Tool

A structured observation tool and field notes were used. The observation tool developed by Ding et al. (2017), which has been applied in other acute care contexts (Gillespie et al. 2020), was used with permission. The original observation tool was modified to enhance contextual relevance for the Sri Lankan setting, based on feedback from the second author (SS), a general surgeon familiar with local clinical practice. The observation tool has three sections. Section one includes variables related to nurses' demographic and professional data. Section two includes variables such as surgical wound type (simple acute, complex acute) (Berríos‐Torres et al. 2017; Ferreira et al. 2006), wound classification (clean, clean contaminated, contaminated, dirty/infected) (Onyekwelu et al. 2017), number of post‐operative days, surgical site, analgesia requirement assessment and dressing type used. Section three comprises seven core domains focusing on activities derived from clinical practice guidelines (CPGs) aimed at surgical wound management (Table S1). Two core domains in the original tool relating to the management and documentation of abnormal wounds, were excluded as they did not apply to the Sri Lankan nursing context. For each item in this section, observers selected one of three possible responses: ‘Yes,’ ‘No’ or ‘Not applicable'. Field notes were recorded as free text and described contextual details relevant to the wound care episodes.

#### 4.3.2 Chart Audit Tool

The authors developed a contextually appropriate chart audit tool based on the literature and previous work in this area. The chart audit tool consisted of 22 items, including patients' demographic and clinical data such as age, gender, type of surgery, comorbidities, admission diagnosis and surgical procedure performed. It also captured and documented postoperative care‐related data, including prescribed dressing solutions, dietitian visits during hospitalisation and any surgical wound complications.

The data collection tools were pilot tested on five observed wound care episodes. Based on the pilot, ‘nurses' notes’ was added as an item under documents used to record the wound care process in the observation tool. Data from the pilot test were excluded from the final sample. Then, tool inter‐rater reliability was tested with three observers independently completing direct observations of the same 20 wound care episodes, standing at different observation points within the room, from which 92% agreement was achieved. Subsequently, the observers independently audited the patient's medical record, achieving 94% agreement between auditors. Discrepancies between observers were discussed and resolved through consensus; when consensus could not be reached, the second author was consulted.

### 4.4 Data Collection

Data collection was undertaken between June and August 2025 by the first author, a senior nursing academic and two trained research assistants (RAs) who were registered nurses with a four‐year nursing degree from a recognised university in Sri Lanka. The RAs were specifically recruited and trained for this study by the first author, 1 week before participant recruitment. The first author closely supervised the data collection undertaken by the RAs through reviewing the data collection tools at the end of each day. A data dictionary was used to enable consistency in the observations made by each observer and reduce interobserver bias. The second author met regularly with the first author to discuss any issues identified during observations and audits (e.g., interpreting handwritten patient records and mapping documented information to predefined audit items). These were clarified through discussion and communicated to the other two observers to ensure consistency.

Each observer was assigned to a maximum of five wards based on ease of access (e.g., wards located in the same building). The observers spent 2 days during wound care rounds on each ward to familiarise themselves with the staff and surgical wound care routines before data collection. This allowed them to establish rapport with the recruited nurses and made them feel relaxed in the presence of the observer. Observers rotated across their allocated wards according to each ward's operative days, ensuring equitable coverage by visiting every ward at least twice a week, typically on one or two days following its scheduled surgeries.

On each data collection day in an included ward, observers met with the nurse in charge to identify bedside nurses assigned for wound dressing. Observers then approached the designated nurses, explained the study purpose and provided an information sheet containing further details. Written informed consent was obtained from nurses during initial recruitment, and consent was reconfirmed verbally before each repeated observation. Observers then liaised with the assigned nurses to identify eligible postoperative patients requiring dressing changes. Patients were approached before their dressing change procedure. A separate information sheet was provided for further details, and written informed consent was obtained from all patients to observe their wound care episodes and to collect details from their medical records.

Observations of a wound care episode began when the nurse performed the first occasion of HH and explained the procedure to the patient and concluded after the completion of the final HH occasion and documentation. During observations, observers were positioned discreetly to avoid direct visibility to nurses while maintaining a clear view of patient and wound care activities. After observing all patients recruited from a particular ward, the medical record audits were completed. Field notes were recorded to give additional contextual details during each of the 250 wound care episodes observed. Observations and chart audits were conducted over 11 weeks on weekdays between 08:00 and 12:00 h, representing about 220 h of observation.

### 4.5 Data Analysis

All observation tools were jointly reviewed by the observer team on the day of data collection to ensure completeness. Open‐ended responses were standardised where appropriate, and the cleaned data were entered independently into IBM SPSS Statistics for Windows (version 31) and verified by the second author. Nurses' demographics were obtained by aggregating repeated observations using a unique nurse identification number. Descriptive statistics were used to calculate absolute (*n*) and relative frequencies (%), means and standard deviations (SD) or medians, interquartile ranges (reported as upper and lower quartiles) and ranges, as appropriate to the level of data and its distribution. Some items in the observation tool (Table 4) were not relevant to every wound care episode, which meant the total number of responses varied across sections. For analysis, percentages were calculated using only valid responses, i.e., those marked ‘Yes’ or ‘No.’ Responses coded as ‘Not applicable’ were not included in the denominator to ensure that percentages reflected only applicable observations. Field notes were used to augment and contextualise the analysis.

### 4.6 Ethical Considerations

This study was approved by the Ethics Review Committee of the respective hospital in Sri Lanka (AAJ/ETH/COM/2025/April) and the Griffith University Human Research Ethics Committee (2025/323). Participants could withdraw from the study at any time. Unique identification numbers were assigned to participants to maintain anonymity. A separate, securely maintained logbook linking nurse participant identities to unique identification numbers was used to facilitate repeated observations. Following ethics approval, permission was obtained from the Chief Nursing Officer to recruit nurses and from consultants of all general surgical wards to recruit patients.

## 5 Results

We observed 250 surgical wound care episodes across 13 general surgical wards. A total of 63 RNs were observed performing wound care. The number of wound care episodes per nurse ranged from 1 to 10, with a median of 3 episodes (IQR = 2–5). The demographic and professional characteristics of the nurse participants are summarised in Table 2. The sample of nurses was predominantly female (56, 88.9%). Nurses' age, years of nursing experience and years of surgical experience demonstrated strong positive skewness (age: 1.7; nursing experience: 1.9; surgical experience: 2.7). Most nurses held a diploma in nursing qualification (*n* = 56, 88.9%). Nearly 60% (*n* = 37, 58.7%) of nurses reported having no prior training related to wound care or infection prevention.

**TABLE 2:** Nurses' demographic and professional characteristics (*N* = 63).

Variable	*N* (%)
Age (years)	
Median (IQR; Range)	28.0 (26.8–30.0; 25.0–39.0)
Sex	
Male	7 (11.1)
Female	56 (88.9)
Nursing experience (years)	
Median (IQR; Range)	1.5 (0.17–4.0; 0.7–14.0)
Surgical experience (years)	
Median (IQR; Range)	0.4 (0.2–2.6; 0.02–14.0)
Designation^a^	
Grade I	1 (1.6)
Grade II	7 (11.1)
Grade III	55 (87.3)
Highest educational qualification	
Diploma	56 (88.9)
Degree	7 (11.1)
Previous training on surgical wound care/infection prevention	
Yes	26 (41.3)
No	37 (58.7)

^a^ Grade classification is based on the years of experience; Grade III: ≤ 5 years; Grade II: 6–12 years; Grade I: 13–20 years; Supra‐grade: ≥ 20 years (didn't involve in dressing change during the observation period).

A total of 250 patients who received wound care from nurses were included in the sample. Of these, 160 (64%) were in‐patients, and 90 (36%) were out‐patients. The median number of days since surgery was 5 (IQR = 2–8; range = 1–28), and the majority (*n* = 176, 70.4%) were > 48 h post‐surgery. Nearly half of all surgeries (*n* = 118, 47.2%) involved gastrointestinal procedures. A summary of patient and surgery‐related characteristics is summarised in Table 3.

**TABLE 3:** Patient and surgery‐related characteristics (*N* = 250).

Variable	*N* (%)
Age (years)	
Mean (SD; range)	49.3 (16.5; 18–85)
Sex	
Male	138 (55.2)
Female	112 (44.8)
Presence of comorbidities^a^	
None	111 (44.4)
Cardio‐pulmonary diseases	91 (36.4)
Diabetes mellitus	76 (30.4)
Cancer	53 (21.2)
Seen by a dietitian during hospitalisation	
Yes	31 (12.4)
No	219 (87.6)
Surgery urgency	
Elective	208 (83.2)
Emergency	42 (16.8)
Surgical procedure classification	
Gastrointestinal	118 (47.2)
Skin, soft tissues and breast	58 (23.2)
Musculoskeletal/limb	32 (12.8)
Head, neck and face	29 (11.6)
Genito urinary	13 (5.2)
Wound type	
Simple acute	165 (66.0)
Complex acute	85 (34.0)
Wound classification	
Clean	97 (38.8)
Clean‐contaminated	79 (31.6)
Contaminated	30 (12.0)
Dirty/infected	44 (17.6)
Wound complications	
None	186 (74.4)
SSI	23 (9.2)
Pre‐existing infections^b^	22 (8.8)
Other complications^c^	19 (7.6)
Antibiotics given	
Yes	225 (90.0)
No	25 (10.0)

Abbreviation: SSIs, Surgical site infections.

^a^ More than one comorbidity documented in medical records; cardio‐pulmonary diseases included are ischaemic heart disease, peripheral vascular disease, renal disease, hypertension and chronic obstructive airways disease.

^b^ Pre‐existing infections include abscess, gangrene or septic limbs.

^c^ Other complications include Surgical wound dehiscence (SWD) (6), bleeding (5), hematoma (2), seroma (2), trauma (1), periwound maceration (1), SSI with bleeding or SWD (2).

### 5.1 Observed Dressing Changes and Removal Practices

Table 4 shows the observed dressing change and removal practices by nurse participants. Compliance with hand hygiene (HH) was greater before dressing changes (146/250, 58.4%) compared with after dressing changes (108/250, 43.2%). However, HH after removing the old dressing was observed on only 21 (17.2%) occasions.

**TABLE 4:** Observed dressing change and removal practices.

Recommended practice	Total, *n* (100%)	Yes, *n* (%)	No, *n* (%)
Leave original dressing for at least 48 h	93*	63 (67.7)	30 (32.3)
b HH before dressing change using soap and water or alcohol gel	250	146 (58.4)	104 (41.6)
c Remove the old dressing with a gloved hand	125*	114 (91.2)	11 (8.8)
d HH after removing the old dressing	122*	21 (17.2)	101 (82.8)
e Change gloves after removing the old dressing	115*	114 (99.1)	1 (0.9)
f Aseptic fields	250	128 (51.2)	122 (48.8)
g Environmental control	250	130 (52.0)	120 (48.0)
h Wear other protective equipment (except gloves)	250	249 (99.6)	1 (0.4)
i HH after dressing change using soap and water or alcohol hand rub	250	108 (43.2)	142 (56.2)

Abbreviation: HH, hand hygiene.

* Totals did not equal 250 due to ‘not applicable’ responses.

Figure 1 summarises the use of gloves and other personal protective equipment (PPE) during wound care episodes. Most nurses used sterile gloves for wound cleansing (*n* = 211, 84.4%) and applying a new dressing (*n* = 250, 100%).

**FIGURE 1:**
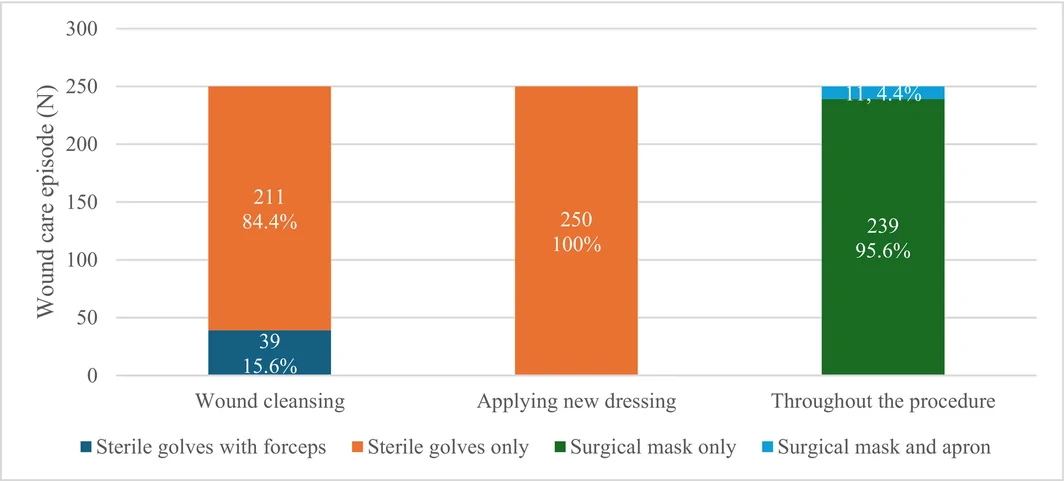
Glove and other personal protective equipment (PPE) use. [Colour figure can be viewed at wileyonlinelibrary.com]

Observations indicated that 45.2% (*n* = 113) of nurses performed one or two additional procedures beyond incision dressing change (Figure 2). The most common additional activities performed by nurses during dressing management were drain care (*n* = 51, 20.4%) and suture or clip removal (*n* = 46, 18.4%). In addition, surgical mask use during dressing procedures was observed in 95.6% (*n* = 239) of occasions.

**FIGURE 2:**
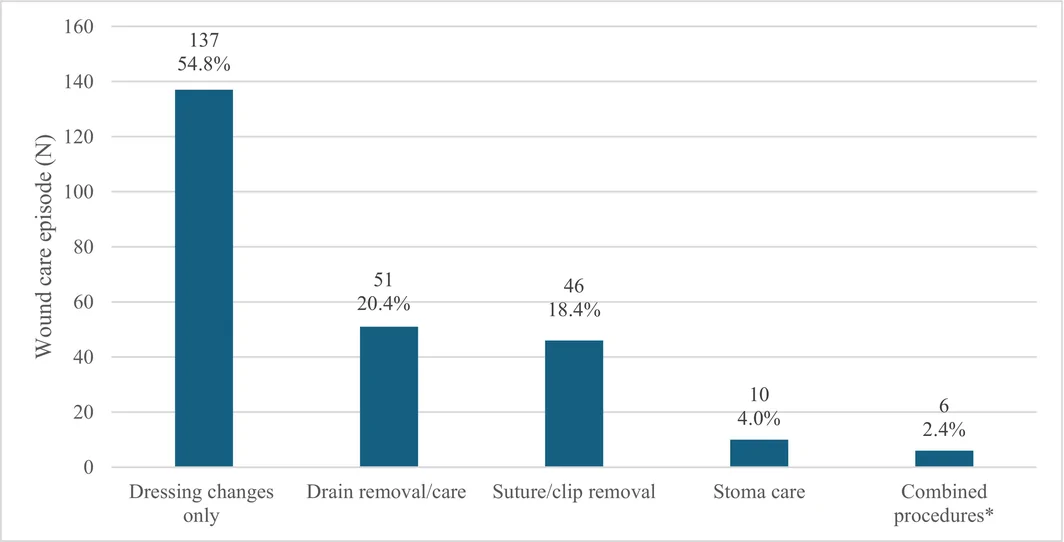
Observed activities undertaken during dressing management. *Combined procedures include drain care with suture/clip removal or stoma care. [Colour figure can be viewed at wileyonlinelibrary.com]

### 5.2 Observed Wound Cleansing and Dressing Solution Use

Wound management decisions, including wound cleansing and dressing solutions, were primarily made by the medical team, and nurses implemented these decisions when prescribed. However, wound cleansing or dressing solutions were prescribed in only 18 of 165 (10.3%) simple acute wounds and 37 of 85 (43.5%) complex acute wounds. In the absence of specific instructions, nurses followed routine ward practices. However, field notes indicate that routine ward practices varied across different wards.

For six wound care episodes, no specific orders were given by the doctor, so the nurse used isopropyl alcohol [rubbing alcohol] to cleanse the simple incision line and normal saline for open surgical wounds. (Field note 38, Ward I)

The nurse used normal saline for cleansing all surgical wounds and applied dry dressings for simple surgical incisions. (Field note 8, Ward B)

Cleansing of wounds within 48 h post‐surgery using normal saline was observed in only 43 of 74 (58.1%) wound care episodes. A total of 135 nurses (54%) were observed using isopropyl alcohol for wound cleansing, regardless of the wound's duration (Table S2). Most Nurses (*n* = 194, 77.6%) cleansed the wound from the least contaminated to the most contaminated area. The use of a single gauze pad for each stroke and drying the wound after wound cleansing was observed in only 21 (8.4%) and 13 (5.2%) episodes, respectively. Dry dressings were the most frequently applied for both wound types: 89.1% (*n* = 147/165) for simple acute wounds and 51.8% (*n* = 44/85) for complex acute wounds (Table S3). Field notes indicated that variations in practices were influenced by resource limitations.

The nurse used one gauze pad to clean inside and outside of the wound, explaining that this was done to conserve sterile supplies. (Field note 28, Ward B)

The nurse explained to the patient that povidone iodine was unavailable and used a dry gauze dressing instead, advising, ‘If you can buy the solution, use it when you change the dressing at home’. (Field note 96, Ward I)

### 5.3 Observed Patient Education Practices

Most patients who were within 48 h post‐surgery (*n* = 69/74, 93.2%) did not receive information on recommendations for showering safely after 48 h. Overall, wound care education, including how to manage wounds after discharge and follow‐up wound care, was observed in only 22% (*n* = 55) of episodes. Field notes revealed that nurses delayed patient education until discharge.

The nurse informed a patient who was being discharged on the same day that instructions would be given later by the discharge nurse before going home. (Field note 117, Ward K)

### 5.4 Observed Wound Assessment and Documentation Practices

All nurses observed used direct visual observation to assess the wound and did not assess pain levels during any wound care episode. Nurses rarely documented wound characteristics. Documentation of dressing change activities was also limited, and any notation by nurses regarding wound care was observed only in 53 (21.2%) wound care episodes. Out of these, inpatient wound care was documented in either patients' medical records (*n* = 33/160, 20.6%) or dressing charts (*n* = 10/160, 6.3%). These dressing charts were ward‐specific and implemented exclusively for patients with surgical wound complications who required prolonged post‐operative stays, with considerable variation in their format across wards. The outpatients' wound care documentation was recorded on the patients' discharge summary document (*n* = 10/90, 12.2%). Field notes suggest that most documentation by nurses lacked details.

The nurse only ticked the doctor's order for dressing changes, either on the inpatients' medical record or on the outpatients' discharge card and recorded these as dressing changes or suture removals completed, along with the date. (Filed note 117, Ward I)

### 5.5 Contextual Observations (Drawn From Field Notes)

Wound care was generally performed at the bedside for inpatients. Of the 13 wards, dressing changes of outpatients were done in only seven wards. Priority was given to outpatient wound management activities: *At 9:30 a.m., nurses completed outpatient dressings before starting inpatient dressing rounds (Field note 35, Ward B)*. It was reported that some wards lacked sufficient facilities for outpatient dressing changes, as described in this field note:

Nurse escorted outpatients to a vacant bed in the ward and performed the dressing there. (Field note 48, Ward K)

Wound assessment and documentation tasks were primarily led by doctors during routine ward rounds. They recorded significant wound‐related assessments in patients' medical records. Clinical decisions regarding wound management were made during ward rounds by the medical team. Nurses assigned to dressing changes and the nurse in charge often attended these ward rounds to receive medical opinions on wound management. Wound dressing procedures are usually performed by nurses afterwards. *The old dressings were removed and assessed during the surgeons' ward round, and dressing rounds commenced afterwards at 10:00 a.m. (Field note 38, Ward M)*. When abnormalities were identified during dressing changes, nurses notified the medical officer and subsequent management decisions were made by the medical team.

The responsibility for performing all required dressing changes was assigned to two nurses on the morning shift, indicating a high nurse‐to‐patient workload:

Two nurses were responsible for performing dressing changes for 15 outpatient and 10 inpatient surgical wounds, in addition to a substantially higher number of chronic wounds. (Field note 192, Ward K)

Nurses were observed to follow ‘aseptic technique’ during the dressing change procedure, and the delegated second nurse was supposed to be the assistant to maintain sterility throughout the dressing round. However, nurses had to seek the help of care assistants or student nurses because of staff shortages. There were instances where nurses had to improvise dressing changes alone due to high patient care demands.

The nurse completed the outpatient dressings alone because the assisting nurse was called to assist with the IV medication trolley. (Field note 67, Ward G)

Nurses' documentation related to wound care was primarily used to support continuity of care rather than to guide treatment decisions. Body temperature was the most consistently recorded assessment by nurses: *At the patient's bedside, temperature chart prominently displayed the surgical procedure and postoperative days from the day of surgery (Field note 89, Ward J)*. There was no standardised surgical wound management protocol or wound assessment tools in any of the wards where observations were conducted.

## 6 Discussion

Our findings highlight that nurses' practices did not consistently align with CPG recommendations, with unwarranted variations in dressing management practices, HH adherence, patient education, wound assessment and documentation. Furthermore, wound care responsibilities were delegated primarily to the junior nurses with limited experience and training. Nurses' clinical decisions during surgical wound management were observed to be multifactorial and influenced by organisational constraints, including limited resources and high workload demand. Nevertheless, there is potential for these practices to be improved even in resource‐constrained settings by targeted training on evidence‐based practice, introducing wound assessment and documentation tools, and standard wound management protocols that involve a multidisciplinary team, including doctors.

In Sri Lanka, dressing change is taught as an aseptic procedure across both diploma and degree level nursing programs using a single sterile dressing packet for each patient, with steps designed to isolate the wound and its immediate surroundings from the dressing field to minimise contamination risk (Gillespie and Fenwick 2009). During student nurses' training, these procedures are taught and evaluated in controlled environments that reinforce ideal standards (Msosa et al. 2022). However, once nurses enter real‐world clinical settings, resource limitations, time pressures and organisational constraints make these ideal practices difficult to maintain (Monaghan 2015). In our sample, which primarily comprised nurses with limited post‐registration experience and prior training, these challenges may have been further intensified, leading to unwarranted variations in practice.

The observed gaps in adherence to CPGs have important implications for patient safety and surgical outcomes. Poor wound care practices increase the risk of surgical wound complications, including SSIs, delayed healing and higher health care costs (Costabella et al. 2023). A clearly defined wound and dressing field, HH and wearing of gloves and other PPEs are considered important during wound dressing (Gillespie and Fenwick 2009). Consistent with our findings, observational studies from developed countries also report notable gaps in nurses' compliance with HH practices, especially after dressing changes (Ding et al. 2017; Gillespie et al. 2020).

Another concerning observation was the use of cleansing solutions and dressings on surgical wounds. Despite contemporary guidelines recommending sterile normal saline for surgical wound cleansing (National Institute for Health and Care Excellence 2019), we observed nurses using isopropyl alcohol on wounds healing by primary intention when no solution was prescribed. Alcohol‐based solutions are indicated for preoperative skin preparation (Hemani and Lepor 2009), and their application on non‐intact skin can damage newly forming tissue, delay healing and cause discomfort (Rabenberg et al. 2002). This highlights a critical knowledge gap. Similarly, our observations of the use of dry dressings appear to be influenced by both knowledge gaps and resource constraints. Consistent with studies from developed countries (Altaweli et al. 2023; Gillespie, Chaboyer, Allen, et al. 2014), nurses demonstrated limited understanding of the need to maintain a moist environment for optimal wound healing, suggesting this is not unique to developing countries like Sri Lanka. However, this challenge is magnified in low‐resource settings where infection control is critical. These practices may also be partially explained by the absence of local policies or standard wound care protocols in this study setting, leading to reliance on routine practices.

Another key issue observed was the lack of patient education on wound care and recovery. Overall, education on wound care and follow‐up was not provided at the bedside during dressing changes, despite evidence that timely, individualised education improves adherence and reduces surgical wound complications (Tobiano et al. 2023). While inpatient education in our setting is often reserved for discharge, this practice overlooks opportunities to reinforce wound care knowledge during dressing changes.

Although documentation is recognised as a professional responsibility and essential for the continuity of care (Demsash et al. 2023), the limited wound assessment and documentation observed among nurses in our sample is concerning. Wound characteristics and care processes were rarely documented, and standard wound assessment tools were not available, resulting in assessments being largely observational, unstructured and lacking essential details. This poses serious risks to continuity of care and patient safety. However, these findings likely reflect systemic constraints, as evidenced by contextual observations of high workload demands and nurses prioritising direct patient care. Similar gaps have been reported in other lower‐middle‐income countries (Do et al. 2021; Oseni and Adejumo 2014). However, studies from high‐income countries also report inconsistencies, mainly in incomplete entries on wound assessment charts (Ding et al. 2017; Gartlan et al. 2010; Gillespie, Chaboyer, Kang, et al. 2014; Gillespie et al. 2020). Implementing structured tools as well as allocating sufficient nursing staff as appropriate can minimise these gaps by promoting consistency, improving communication among professionals and ensuring timely identification of complications (Do et al. 2022).

In addition to nurse‐related and resource limitations, systemic gaps were evident in our setting. Along with the aforementioned absence of wound assessment and documentation tools, standard wound management guidelines were lacking. Moreover, observed variation in adherence to existing CPGs underscores the importance of adapting them to local resource constraints and care realities. Standardised wound management protocols ensure consistency among professionals. Evidence suggests that a lack of clear protocols contributes to inconsistent care, delayed healing and increased risk of SSIs (Leaper et al. 2015). Addressing these gaps through institutional policies, standardised protocols tailored to low‐resource settings and continuous education is essential to ensure safe, effective, and sustainable wound care.

### 6.1 Strengths and Limitations

To the best of our knowledge, this is the first observational study conducted in a low‐resource setting within this field, providing novel insights into nurses' clinical practice. Unlike most previous studies conducted in low‐resource settings, which have primarily relied on self‐reported surveys (Famakinwa et al. 2014; Gizaw et al. 2022; Tegegn et al. 2023), this study employed direct observation of wound care practice. This notable strength enabled the capture of actual practices rather than retrospective, self‐reported behaviours. The inclusion of chart audits and detailed field notes added to a comprehensive understanding of the clinical context. Nurses' adherence to best practices was also assessed using an evidence‐based tool, with data collection conducted by trained observers and adequate inter‐rater reliability established, ensuring methodological rigour.

To minimise the Hawthorne effect, the research team spent additional time in the wards before data collection, avoided disclosing specific aspects under observation and positioned observers discreetly; however, this effect could not be completely eliminated. Another limitation of our study is that the sample was drawn from a single hospital, which may differ from other hospitals. Nevertheless, the site represents the largest tertiary care hospital in Sri Lanka, with a 3000‐bed capacity and general surgical wards, offering a broad representation of practices within a major healthcare institution. Despite these limitations, the study provides robust and contextually rich evidence on surgical wound care practices in a low‐resource setting, forming an important foundation for future research and interventions aimed at improving surgical care quality.

### 6.2 Recommendations for Further Research

This study underscores the need for targeted interventions to improve the quality and consistency of surgical wound care practices by nurses, as well as the coordination of care within the multidisciplinary team. Future research should explore the factors that influence nurses' wound care practices, including their current knowledge, barriers and facilitators to implementing evidence‐based practice in ward settings. Studies involving multiple hospitals across both public and private sectors are recommended to enhance the generalisability of findings. Designing and testing context‐specific interventions that address both knowledge gaps and resource limitations in low‐resource environments is essential. Additionally, research should examine the effectiveness of structured training programs for nurses, the implementation of standardised wound assessment tools and the impact of improved documentation systems on patient outcomes. Both longitudinal and interventional studies of this type are needed to assess the impact of practice support strategies and infrastructure, and how sustained policy changes and resource allocation influence wound care practices over time.

### 6.3 Implications for Policy and Practice

This study highlights critical gaps in nurses' surgical wound care practices, underscoring the need for systemic improvements in this context. Targeted training programs for RNs, particularly junior staff, should focus on evidence‐based wound management and strategies for adapting guidelines in resource‐limited situations. At the policy level, health authorities should develop and implement context‐sensitive standardised wound care protocols, adequate resource provision and clear delineation of professional roles of nurses and doctors in wound management to reduce unwarranted variability. Additionally, fostering multidisciplinary collaboration between nurses and doctors can improve communication and continuity of care. These measures can collectively enhance the quality, consistency and safety of surgical wound care in low‐resource settings. Establishing routine SSI surveillance systems in Sri Lanka, where SSI incidence data are not reported, should be prioritised to support monitoring of patient outcomes and evaluation of future quality improvement initiatives.

## 7 Conclusion

Our findings suggest that nurses' overall adherence to CPGs was limited, and there was an unwarranted practice variation in nurses' surgical wound care practices. Such variations can be attributed to several factors, including gaps in knowledge on evidence‐based surgical wound management and delegation of wound care tasks to inexperienced nurses with limited training. Further, some systemic issues, such as resource constraints, limited wound assessment tools and standard wound management protocols, further exacerbate these inconsistencies. Interdisciplinary team training and introducing context‐specific, standard surgical wound care protocols, including standard wound assessment and documentation, may contribute to improvements in nurses' surgical wound care practices.

## Author Contributions

Gayani Priyangika Gamage: Writing – original draft, writing – review and editing, methodology, investigation, formal analysis, data curation, conceptualisation. Sanjeewa Seneviratne: Writing – review and editing, supervision, methodology, conceptualisation. Josephine Lovegrove: Writing – review and editing, supervision, methodology, conceptualisation. Georgia Tobiano: Writing – review and editing, supervision, methodology, conceptualisation. Brigid Mary Gillespie: Writing – review and editing, supervision, methodology, formal analysis, conceptualisation.

## Funding

This research was supported by the Griffith University Postgraduate Research Scholarship (GUPRS), the Griffith University International Postgraduate Research Scholarship (GUIPRS) and Griffith University Higher Degree Research (HDR) funding. The funders had no role in the study design, data collection, analysis, interpretation or preparation of the manuscript.

## Conflicts of Interest

The authors declare no conflicts of interest.

## Supporting information


**Table S1:** CPG recommendations for SSI prevention in surgical wound care.
**Table S2:** Observed wound cleansing practices.
**Table S3:** Prescribed and applied wound dressing products.

## Data Availability

The data that support the findings of this study are available on request from the corresponding author. The data are not publicly available due to privacy or ethical restrictions.
