# Supplementary material for: Surgical Wound Care Practices of Nurses in a Low‐Resourced Setting: An Observational Study
Source: J Clin Nurs. 2026 Jul 12;35(12):5485–95. doi: 10.1111/jocn.70445 (PMC13744233; doi:10.1111/jocn.70445)
Supplement: Supplementary file 1 [file JOCN-35-5485-s001.docx]

***Supplementary Table 1: CPG recommendations for SSI prevention in surgical wound care***

| Core criterion | CPG recommendation | References |
| --- | --- | --- |
| Dressing management | - Leave dressing intact for at least 48 hours postop - Hand washing before dressing change, using soap and water, or alcohol gel - Remove old dressing with gloved hands. - Wash hands after removing the old dressing - Change gloves after removing the old dressing - Use clean gloves in non-touch technique (gloved hand does not touch wound, and/or dressing) - Use sterile gloves if gloved hand touching the wound and/or dressing is unavoidable - Aseptic fields - Dress wound using non-touch technique (gloved hand does not touch wound) - Environmental control: no other avoidable infection risk factors (such as bed making, patients using commodes, or urinal bottles with urine in them) - Wear other protective equipment if contamination is expected, including eye protection - Hand washing after dressing change using soap and water, or alcohol gel | National Institute for Health and Care Excellence (2019), Berrios-Torres et al. (2017) |
| Wound cleansing | - Use sterile saline for wound cleansing from post-op to 48 hours after surgery if dressing needs to be changed - Use tap water for wound cleansing after 48 hours, and if the wound has separated or has been surgically opened to drain pus | National Institute for Health and Care Excellence (2019) |
| Using topical antimicrobial agents | - Used topical antimicrobial agents on surgical wounds healing by primary intention | National Institute for Health and Care Excellence (2019)  World Health Organisation (2018) |
| Patient education | - Advise patients that they may shower safely 48 hours after surgery - Educate the patient on how to care for the wound after discharge and follow up on wound care | National Institute for Health and Care Excellence (2019), Berrios-Torres et al. (2017) |
| Wound assessment methods | - Direct observations - Wound assessment tools | Berrios-Torres et al. (2017) |
| Documentation of wound care process: Where is it documented? | - On treatment sheets/Bed Head Ticket - Other tools | Haesler and Carville (2023) |
| Wound documentation contents | - Type of wound - Duration of wound - Location of wound - Dimension of wound (length x width x depth) - Clinical characteristics of wound bed (red, pink, yellow, black, undermined) - Wound edge appearance (callus and scale, maceration, erythema, edema) - Peri wound appearance - Exudate type and colour - Infection: signs and symptoms of infection documented. - Wound pain - Presence of foreign bodies - Prior wound treatments and their therapeutic outcome | National Institute for Health and Care Excellence (2019)  Berrios-Torres et al. (2017); Haesler and Carville (2023) |

**Supplementary Table 2: Observed wound cleansing practices**

| Cleansing solution | | | ≤ 48 hours  (n=74) | >48 hours  (n=176) |
| --- | --- | --- | --- | --- |
| Normal saline | 43 (58.1) | | | 71(37.5) ^a^ |
| Isopropyl alcohol | | 28 (37.8) | | 100(51.7) ^b^ |
| Isopropyl alcohol (incision)+ normal saline (periwound) | | 2(2.7) | | 5(2.8) |
| 3% saline ^c^ | 1(1.4) | | | - |
| Total | 74(100.0) | | | 176(100.0) |
| ^a^ Includes 5 patients who had a shower before dressing change; ^b^ Includes 9 patients who had a shower before dressing change; ^c^ After a shower before dressing change | | | | |

***Supplementary Table 3: Prescribed and applied wound dressing products***

| Simple acute (N=165) | Prescribed, N (%) | | | Applied, N (%) |
| --- | --- | --- | --- | --- |
| Dry dressing | 13 (7.9) | | | 147 (89.1) |
| Povidone iodine | 2 (1.2) | | | 15 (9.1) |
| Other prescribed (Fusidic cream, normal saline) | 2 (1.2) | | | N/A |
| Other applied (No dressing, SSD, 3% NaCl) | N/A | | | 3 (1.8) |
| Not reported | 148 (89.7) | | | N/A |
| Complex acute (N=85) | **Prescribed, N (%)** | | | **Applied, N (%)** |
| Povidone iodine | 10 (11.8) | 23(27.1) | | |
| Milton solution (0.25%) | 7 (8.2) | 5 (5.9) | | |
| Dry dressing | 5 (5.9) | 44 (51.8) | | |
| Fender plus (hydrogel) | 3 (3.5) | 2 (2.4) | | |
| Hypertonic/ 3% NaCl | 4 (4.7) | 4 (4.7) | | |
| Normal saline | 4 (4.7) | 4 (4.7) | | |
| Other prescribed (Bioplus, Fusidic cream, 1% acetic acid, Metrogel) | 4 (4.7) | N/A | | |
| Other applied (Bioplus, Vaseline gauze, 2% sodium fusidate dressing) | N/A | 3 (3.5) | | |
| Not reported | 48 (56.5) | | N/A | |
| Abbreviations: N/A= Not Applicable; SSD = Silver sulphadiazine; NaCl= Sodium chloride | | | | |

Berrios-Torres, S. I., Umscheid, C. A., Bratzler, D. W., Leas, B., Stone, E. C., Kelz, R. R., Reinke, C. E., Morgan, S., Solomkin, J. S., Mazuski, J. E., Dellinger, E. P., Itani, K. M. F., Berbari, E. F., Segreti, J., Parvizi, J., Blanchard, J., Allen, G., Kluytmans, J., Donlan, R., . . . Healthcare Infection Control Practices Advisory, C. (2017). Centers for Disease Control and Prevention Guideline for the Prevention of Surgical Site Infection, 2017. *JAMA Surg*, *152*(8), 784-791. <https://doi.org/10.1001/jamasurg.2017.0904>

Haesler, E., & Carville, K. (2023). *Australian Standards for Wound Prevention and Management*. Cambridge Media on behalf of the Australian Health Research Alliance, Wounds Australia, and WA Health Translation Network. <https://woundsaustralia.org/int/woundsaus/uploads/Publications/Standards%20and%20Guidelines/Australian%20Standards%20for%20Wound%20Prevention%20and%20Management%204th%202023.pdf>

National Institute for Health and Care Excellence, N. (2019). Surgical site infections: prevention and treatment. 31. <www.nice.org.uk/guidance/ng125>

World Health Organisation, W. (2018). *Global Guidelines for the Prevention of Surgical Site Infections* (2nd ed.). World Health Organization, (WHO).
